# Heat shock protein Gp96 (Grp94) in malaria: functional insights at the host-parasite interface and therapeutic perspectives

**DOI:** 10.3389/fcell.2026.1760166

**Published:** 2026-03-26

**Authors:** Djibaba Djoumoi, Abou Abdallah Malick Diouara, Mamadou Diop, Cheikh Momar Nguer, Babacar Mbengue, Fatou Thiam

**Affiliations:** 1 Groupe de Recherche Biotechnologies Appliquées & Bioprocédés Environnementaux (GRBA-BE), Laboratoire Eau, Energie, Environnement et Procédés Industriels (LE3PI), École Supérieure Polytechnique, Université Cheikh Anta Diop de Dakar, Dakar, Senegal; 2 Service d’Immunologie, Faculté de Médecine, de Pharmacie et d’Odontostomatologie, Université Cheikh Anta DIOP, Dakar, Senegal

**Keywords:** Gp96, Grp94, heat shock proteins, malaria, *PfGp96*, *Plasmodium falciparum*, therapeutic targeting

## Abstract

Heat shock protein Gp96 (also known as Grp94 or endoplasmin) is the endoplasmic reticulum (ER)-resident paralog of the Hsp90 family and a central regulator of ER proteostasis and immune receptor biogenesis in mammalian cells. By controlling the folding, quality control, and trafficking of a restricted yet functionally critical set of client proteins, including Toll-like receptors, integrins, and immunoglobulins, Gp96 plays an essential role in innate immunity and inflammatory signaling. In the context of malaria, accumulating evidence suggests that host-derived Gp96 is involved in immune activation and disease severity, notably through its extracellular release under conditions of cellular stress, where it functions as a danger-associated molecular pattern (DAMP). Elevated circulating Gp96 levels have been associated with severe malaria phenotypes, supporting its potential value as a biomarker of host stress and immune dysregulation. In parallel, *Plasmodium falciparum* expresses its own ER-resident Hsp90 homolog, PfGp96, which retains the conserved domain architecture of Hsp90 while exhibiting parasite-specific adaptations, including divergence in ER retention motifs. However, the biological functions, client repertoire, and essentiality of PfGp96 remain poorly defined, and direct evidence supporting its validation as a drug target is currently limited. This review critically synthesizes current knowledge on Gp96 and PfGp96, emphasizing experimentally validated functions, host-parasite interface dynamics, and unresolved knowledge gaps. We discuss the opportunities and challenges of targeting Gp96-related pathways for biomarker development and therapeutic intervention in malaria, while outlining key priorities for future functional and translational research.

## 1 Introduction

Maintenance of cellular proteostasis is essential for cell survival, development, and adaptation to stress (Hohfeld et al., 2021). This process relies on coordinated networks of molecular chaperones that regulate protein folding, prevent aggregation, and ensure quality control within distinct subcellular compartments (Voisine et al., 2010). Among these networks, the heat shock protein 90 (Hsp90) family plays a prominent role due to its involvement in the maturation and stabilization of proteins required for core cellular functions (Shahinas et al., 2013; Chiosis et al., 2023; Karras et al., 2025).

In eukaryotic cells, the Hsp90 family comprises several compartment-specific paralogs that share a conserved structural framework but differ in localization and functional specialization (Hoter et al., 2018). The endoplasmic reticulum (ER)-resident paralog Gp96 (also termed Grp94 or endoplasmin), encoded by *HSP90B1*, is dedicated to ER proteostasis (Marzec et al., 2012; Pugh et al., 2022). Unlike cytosolic Hsp90 isoforms, Gp96 supports to the ER folding and quality control of a restricted but functionally critical subset of secretory and membrane proteins that govern immune receptor biogenesis (Eletto et al., 2010; Hendershot, 2025; Kuzu et al., 2025). Genetic and biochemical studies in mammalian systems have established Gp96 as an essential component of ER quality control and cellular homeostasis (Audouard et al., 2011).

In addition to its intracellular functions, Gp96 has been detected at the cell surface or in the extracellular environment under conditions associated with cellular stress or tissue injury (Martins et al., 2012; Seignez et al., 2017). In these contexts, extracellular Gp96 has been reported to participate in immune-related signaling processes. However, the mechanisms governing its release and the functional consequences of its extracellular presence appear to be context dependent and are not uniformly established across disease settings (Cosin-Roger et al., 2018).

Malaria, caused by *Plasmodium* parasites, is characterized by complex host-parasite interactions accompanied by immune activation, inflammation, and tissue damage, particularly in severe forms of the disease (Pavithra et al., 2004; Gowda and Wu, 2018; Blatch, 2022; Zekar and Sharman, 2023; Li et al., 2025; Meena et al., 2026). A clinical study has reported elevated circulating levels of Gp96 in patients with severe malaria, indicating an association between host cellular stress responses and disease severity (Thiam et al., 2025). Despite its association with disease severity, current evidence remains correlative, and whether extracellular Gp96 directly contributes to malaria pathophysiology or chiefly reflects host ER stress and inflammatory responses remains unresolved.

At the same time, *Plasmodium falciparum* expresses an ER-resident Hsp90 homolog, *PfGp96*, which retains the conserved domain architecture of the Hsp90 family while exhibiting parasite-specific sequence features (Dutta et al., 2022; Muzenda et al., 2025). Although PfGp96 is presumed to contribute to parasite ER proteostasis, its functional network, client repertoire, and essentiality during the parasite life cycle have not yet been comprehensively characterized (Muzenda et al., 2025).

Taken together, current evidence highlights substantial gaps in our understanding of both host Gp96 and parasite PfGp96 in malaria. In this review, we synthesize available data on these ER-resident chaperones in the context of ER proteostasis, immune signaling, and host-parasite interactions relevant to malaria. We explicitly distinguish experimentally validated mechanisms from hypotheses extrapolated from other disease contexts, identify key biological and translational knowledge gaps, and critically discuss the potential, alongside the limitations of Gp96-related pathways as biomarkers or therapeutic targets.

## 2 Methodology

This narrative review was conducted using a structured and transparent literature search strategy aimed at identifying and synthesizing current evidence on the biological functions, pathophysiological roles, and potential therapeutic relevance of the endoplasmic reticulum-resident chaperone Gp96 in malaria (Page et al., 2021).

Electronic searches were performed in PubMed/MEDLINE, Web of Science, Scopus, and Google Scholar, covering publications from database inception to June 2025. Search terms included combinations of “Gp96”, “Grp94”, “HSP90B1”, or “endoplasmin” with “malaria,” “Plasmodium falciparum,” and related terms. These searches were complemented by keywords associated with endoplasmic reticulum stress, heat shock proteins, immune signaling, disease severity, biomarkers, and therapeutic targeting. Reference lists of relevant articles were manually screened to identify additional studies of potential relevance.

Eligible publications included peer-reviewed original research articles, clinical observational studies, *in vitro* and *in vivo* experimental studies, and reviews providing mechanistic insights relevant to Gp96 biology and host–parasite interactions. Articles were excluded if they were not published in English, were conference abstracts without accessible full data, or lacked experimental, mechanistic, or clinical relevance to Gp96. As this work was designed as a narrative review, no formal systematic review protocol or meta-analytic approach was applied.

Given the limited number of studies directly investigating Gp96 in malaria, a narrative synthesis approach was adopted. Evidence was stratified according to study type and level of inference, distinguishing experimental cellular studies, animal models, human clinical data, and indirect evidence derived from non-malarial disease contexts. Particular attention was paid to study design, cohort size, biological matrices analyzed, analytical methodologies, and potential confounding factors when interpreting associations between circulating or extracellular Gp96 and disease severity. Evidence derived from other disease settings was explicitly identified as supportive or hypothesis-generating rather than definitive in the context of malaria.

Importantly, the available literature remains heterogeneous, with limited malaria-specific experimental validation and variability in study design and analytical approaches (Muzenda et al., 2025). These limitations were taken into account when interpreting the data, and areas where evidence remains insufficient or speculative are explicitly highlighted throughout the review.

## 3 Gp96 within the Hsp90 family: positioning a specialized ER chaperone

Hsp90 is a highly conserved molecular chaperone essential for proteome stability in eukaryotic cells (Hu et al., 2022; Singh et al., 2024; Gahlawat et al., 2025). By supporting the folding, maturation, and stabilization of client proteins, Hsp90 regulates fundamental processes such as signal transduction, stress adaptation, and development (Hoter et al., 2018; Monteleone et al., 2025). In eukaryotes, Hsp90 is not a single protein but a small family of paralogs that share a conserved architecture while exhibiting distinct subcellular localizations and functional specializations (Johnson, 2012). This diversification has led to functionally non-redundant Hsp90 isoforms adapted to specific cellular compartments, including the cytosol, mitochondria, and endoplasmic reticulum (ER) (Upadhyay et al., 2025).

Gp96, encoded by *HSP90B1*, is the only Hsp90 paralog localized to the ER lumen (Pugh et al., 2022; Xu and Blagg, 2025). Unlike cytosolic Hsp90 isoforms, which interact with a broad and dynamic range of signaling proteins, Gp96 supports the folding and maturation of a restricted but functionally critical subset of secretory and membrane proteins (Chhabra et al., 2015; Huang et al., 2022). Genetic studies demonstrating embryonic lethality following *HSP90B1* deletion underscore the non-redundant and essential role of Gp96 in ER homeostasis and organismal development, distinguishing it from other Hsp90 paralogs that display partial functional redundancy (Yang and Li, 2005; Audouard et al., 2011).

All Hsp90 family members share a conserved tripartite organization comprising an N-terminal ATP-binding domain, a middle domain involved in client interactions, and a C-terminal dimerization domain (Taipale et al., 2010; Taipale et al., 2012; Singh et al., 2024). Within this conserved framework, Gp96 displays distinctive structural features that reflect its ER specialization. These include a C-terminal ER retention signal, classically KDEL in mammalian cells, and a unique five-amino acid insertion (QEDGQ) in the pre-N-terminal domain that generates a secondary hydrophobic pocket absent from other Hsp90 isoforms (Crowley et al., 2016; Duan et al., 2021). In addition, Gp96 contains a charged linker region with calcium-binding capacity, consistent with adaptation to the ionic environment of the ER lumen (Samy et al., 2021; Amankwah et al., 2024). Together, these features differentiate Gp96 from cytosolic and mitochondrial Hsp90 isoforms and provide a structural basis for isoform-selective targeting (Jia et al., 2025).

**FIGURE 1:**
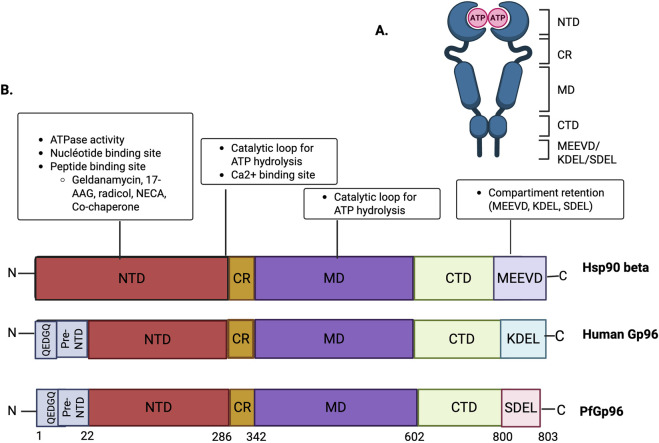
Structural organization and comparative domain architecture of cytosolic Hsp90, human Gp96/Grp94, and Plasmodium falciparum PfGp96. **(A)** ATP-dependent conformational cycle of Hsp90 family members. ATP binding to the N-terminal domain (NTD) promotes dimer closure, followed by ATP hydrolysis and transition to the ADP-bound state. **(B)** Linear domain organization of cytosolic Hsp90, human Gp96/Grp94, and PfGp96. All share a conserved architecture comprising an N-terminal ATP-binding domain (NTD), charged region (CR), middle domain (MD) involved in client interaction and ATP hydrolysis, and a C-terminal dimerization domain (CTD). Cytosolic Hsp90 terminates with the MEEVD motif for TPR co-chaperone recruitment, whereas ER-resident paralogs contain ER retention motifs (KDEL in human Gp96; SDEL in PfGp96). Human Gp96 harbors a five–amino acid insertion (QEDGQ) in the N-terminal region forming a secondary hydrophobic pocket implicated in selective inhibitor binding. PfGp96 retains the canonical framework but displays parasite-specific sequence divergence. Functional elements shown include the ATP-binding site, catalytic loop, Ca^2+^-binding site, and representative ligand-binding regions (e.g., geldanamycin derivatives, 17-AAG, radicicol). SP, signal peptide; Pre-NTD, N-terminal pre-domain region.

In *P*. *falciparum*, the Hsp90 family is conserved and includes an ER-associated paralog, PfGp96. While PfGp96 retains the canonical Hsp90 domain architecture, it exhibits notable sequence divergence from human Gp96. In particular, PfGp96 terminates with an SDEL motif rather than the canonical KDEL sequence, and its precise ER localization and trafficking dynamics remain incompletely resolved (Stofberg et al., 2021) (Figure 1). Comparative analyses suggest that PfGp96 preserves the core Hsp90 fold while displaying parasite-specific adaptations, potentially reflecting the unique proteostatic demands of the parasite ER (Newstead and Barr, 2020). However, PfGp96 remains far less characterized than its mammalian counterpart, and its integration into parasite-specific folding networks has not yet been experimentally defined (Blatch, 2022; Muzenda et al., 2025). Current evidence regarding its essentiality, client repertoire, and regulatory partners in *P*. *falciparum* relies predominantly on sequence-based predictions and comparative analyses, rather than on direct genetic or biochemical validation.

Integrated structural, evolutionary, and localization analyses position Gp96 as a specialized, ER-dedicated Hsp90 paralog that is functionally and spatially distinct from other family members (Hoter et al., 2018; Xu and Blagg, 2025). While its essential role in ER proteostasis, immune receptor biogenesis, and organismal development is well established in mammalian systems (Marzec et al., 2012; Pugh et al., 2022), the extent to which these principles extend to PfGp96 in *P. falciparum* remains unresolved.

Together, these observations highlight critical gaps in our understanding of Gp96-dependent pathways at the host-parasite interface and underscore the need to critically examine the functional roles, regulatory mechanisms, and biological implications of host Gp96 and parasite PfGp96 in malaria, with careful distinction between experimentally validated mechanisms, cautious extrapolations from other systems, and hypotheses that remain to be tested.

## 4 Gp96 in ER proteostasis, immune signaling, and the host-parasite interface in malaria

Gp96 is essential for ER proteostasis through the folding and trafficking of a restricted set of client proteins, including Toll-like receptors, integrins, and immunoglobulins (Table 1) (Marzec et al., 2012; Hoter et al., 2018; Xu and Blagg, 2025). These functions position Gp96 at the interface between ER homeostasis and innate immune signaling. Under cellular stress, Gp96 can be relocalized or released extracellularly, where it has been associated with immune activation (Eletto et al., 2010; Seignez et al., 2017). In malaria, these host-derived roles coexist with a parasite ER paralog, PfGp96, whose biological functions remain poorly characterized (Blatch, 2022; Muzenda et al., 2025).

**TABLE 1:** Functional landscape of Gp96/Grp94: major client proteins, co-chaperones, and stress-related partners.

Category	Protein/Partner	Function/Role	Relationship with Gp96	Relevance to malaria and pathophysiology	References
Canonical client proteins	TLR2, TLR4, TLR9	Innate immune receptors recognizing microbial and parasitic ligands	Gp96 chaperones TLR ectodomains, ensuring proper folding and ER export	Mediate recognition of *Plasmodium* glycosylphosphatidylinositols and hemozoin-DNA; hyperactivation drives inflammation	Liu et al. (2010); Wu et al. (2012); Gowda and Wu (2018); Xu and Blagg (2025)
	Integrins (αLβ2, α2β1, β2)	Leukocyte adhesion, migration, and tissue integrity	Gp96 required for heterodimer assembly and surface trafficking	Dysregulated integrin folding affects immune cell infiltration during malaria	Staron et al. (2010); Pugh et al. (2022)
	Immunoglobulins (IgG, IgM)	Adaptive immune effector proteins	Gp96 facilitates ER folding and secretion via co-chaperone MZB1	Influences antibody secretion and humoral immunity during infection	Rosenbaum et al. (2014); Marzec et al. (2012)
	LRP6	Co-receptor in Wnt/β-catenin signaling	Gp96 ensures proper folding and membrane trafficking	May influence vascular remodeling during infection-induced stress	Duan et al. (2021)
Co-chaperones	CNPY3 (Canopy 3)	ER adaptor for TLR biogenesis	Stabilizes TLR folding intermediates, directs them into the Gp96 cavity	Ensures proper innate immune receptor maturation	Liu et al. (2010); Leu et al. (2011); Marzec et al. (2012)
	ERdj3 (DNAJB11)	Hsp40-family co-chaperone linking BiP to Gp96	Recognizes unfolded glycoproteins and channels them into the ERQC network	May have *Plasmodium*-specific analogs for ER stress regulation	Chen et al. (2017); Sun et al. (2019); Blatch (2022)
	MZB1	B-cell–specific ER co-chaperone	Associates with Gp96 in an ATP-dependent manner during Ig assembly	Stabilizes B-cell secretory function; enhances adaptive immunity	Rosenbaum et al. (2014); Marzec et al. (2012)
	p23, FKBP family (FKBP8)	ATPase-cycle modulators	Fine-tune conformational dynamics and client specificity	Potential auxiliary targets in parasite systems	Hessling et al. (2009); Xu and Blagg (2025)
Extracellular/stress-related interactors	Complement C3	Innate immune effector	Forms complex with extracellular Gp96 under ER stress	Gp96–C3 co-secretion enhances C3 cleavage, fueling pro-inflammatory macrophage phenotype	Seignez et al. (2017); Chaumonnot et al. (2021)
	CD91 (LRP1)	Receptor for extracellular HSPs	Binds secreted Gp96–peptide complexes, mediating cross-presentation	Enhances anti-parasite T-cell responses; hyperactivation linked to cerebral malaria	Jockheck-Clark et al. (2010); Thiam et al. (2025)
	BiP/GRP78	Master ER chaperone for unfolded proteins	Cooperates with Gp96 in substrate capture, transfer, and release	Coordinates ER proteostasis and stress response during infection	Eletto et al. (2010); Zhu and Lee (2015); Haag et al. (2022)

This table summarizes the main interacting partners and client proteins of the ER-resident chaperone Gp96 (Grp94). Gp96 cooperates with specific co-chaperones (CNPY3, ERdj3, MZB1, BiP) to ensure folding of immune and adhesion receptors and modulates extracellular immune signaling through CD91 and complement C3. Dysregulation or extracellular release of Gp96 contributes to inflammatory pathology in malaria, highlighting its dual role as both a biomarker and a therapeutic target.

### 4.1 ER stress responses and Gp96: host versus parasite contexts

**FIGURE 2:**
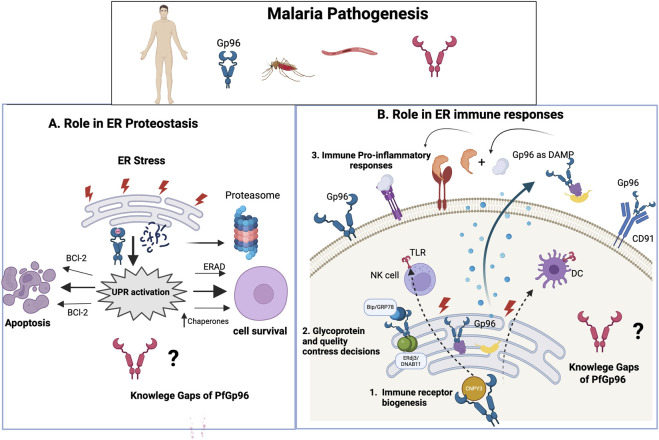
Gp96-Driven ER Proteostasis and Immune Regulation in Malaria: Host Mechanisms and Knowledge Gaps for PfGp96. **(A)** Role in ER proteostasis. During endoplasmic reticulum (ER) stress, Gp96 contributes to unfolded protein response (UPR) activation and ER-associated degradation (ERAD), thereby influencing cell fate decisions. By facilitating protein folding and quality control, Gp96 promotes cellular adaptation and survival. Persistent ER stress may instead trigger apoptosis, partly through modulation of BCL-2 family proteins. The involvement of parasite PfGp96 in ER stress responses and parasite proteostasis remains poorly defined. **(B)** Role in ER immune responses and extracellular signaling. Within the ER, Gp96 is essential for immune receptor biogenesis, including Toll-like receptors (TLRs) and integrins, acting in coordination with ER-resident partners such as BiP/GRP78 (binding immunoglobulin protein), ERdj3/DNAJB11, and CNPY3. Proper glycoprotein folding and quality control shape downstream immune activation. Under stress conditions, Gp96 can be released extracellularly, where it functions as a danger-associated molecular signal and engages CD91 (LRP1), promoting dendritic cell (DC) activation and pro-inflammatory responses. Natural killer (NK) cells and other immune effectors may be indirectly influenced through these pathways. In contrast, the functional network, client repertoire, and immunological role of PfGp96 in malaria remain largely unresolved.

In higher eukaryotes, perturbations of ER homeostasis activate adaptive unfolded protein response (UPR) programs that restore proteostasis or trigger apoptosis under sustained stress (Kaushansky and Kappe, 2015; Hetz et al., 2020; Hetz et al., 2024) (Figure 2). Although Gp96 is not a canonical UPR sensor, it contributes functionally to stress adaptation by maintaining ER folding capacity and supporting ER-associated degradation (ERAD) pathways (Zhu and Lee, 2015; Jeon and Ronai, 2026).

In contrast, *P. falciparum* lacks the canonical UPR signaling machinery found in mammalian cells, as demonstrated by bioinformatic and experimental analyses (Chaubey et al., 2014; Kirchner et al., 2016). Pharmacological induction of ER stress in the parasite does not lead to coordinated upregulation of ER chaperones or ERAD components; instead, it elicits a parasite-specific transcriptional response dominated by AP2 transcription factors, promoting protein export programs and sexual differentiation (Galluzzi et al., 2017; Shang et al., 2022).

Although the parasite experiences substantial oxidative and proteotoxic stress during intraerythrocytic development (Haag et al., 2022), the role of PfGp96 in these adaptive responses remains undefined. To date, no direct genetic or biochemical evidence establishes its essentiality, regulatory mechanisms, or client spectrum in *P. falciparum*.

### 4.2 Gp96 Co-chaperones and ER quality control networks

Within the ER lumen, Gp96 does not function in isolation but operates as part of a highly coordinated chaperone network that ensures substrate specificity, folding efficiency, and quality control (Hendershot, 2025). This network comprises a restricted set of ER-resident co-chaperones that modulate Gp96 activity, client selection, and integration into ER proteostasis pathways (Kuzu et al., 2025).

Among the best-characterized Gp96 co-chaperones is canopy homolog 3 (CNPY3), which is essential for Toll-like receptor (TLR) biogenesis (Liu et al., 2010). CNPY3 acts as a Gp96-specific cochaperone, stabilizing immature TLRs and facilitating their productive folding and trafficking to the cell surface (Leu et al., 2011). Genetic ablation of CNPY3 phenocopies Gp96 deficiency with respect to TLR expression, underscoring their functional interdependence in innate immune receptor maturation (Mutoh et al., 2018).

Gp96 activity is further coordinated with ERdj3/DNAJB11, a member of the Hsp40 family that bridges Gp96 and BiP/GRP78 during glycoprotein folding (Chen et al., 2017; Sun et al., 2019). ERdj3 contributes to substrate transfer and quality control decisions, particularly under ER stress, thereby linking Gp96-dependent folding to broader ER stress response pathways (Huang et al., 2022). In specialized immune cells, MZB1 has been identified as a Gp96-associated factor that selectively supports immunoglobulin assembly and secretion, highlighting cell type-specific adaptations of the Gp96 chaperone network (Rosenbaum et al., 2014; Bhanuse et al., 2025). Collectively, these co-chaperones define a Gp96-centered ER quality control module that is indispensable for immune receptor biogenesis, antibody production, and maintenance of ER proteostasis in mammalian cells (Pugh et al., 2022). This regulatory architecture differs markedly from cytosolic Hsp90 cochaperone systems (Maharaj et al., 2016), reinforcing the concept that Gp96 has evolved a specialized mode of regulation adapted to the ER environment.

In contrast, the existence and functional relevance of comparable co-chaperone systems for PfGp96 in *P. falciparum* remain unknown. Although the parasite encodes homologs of several ER-resident chaperones (Florentin et al., 2020), no experimental evidence has yet demonstrated specific cochaperones that interact with PfGp96 or regulate its folding cycle. Current hypotheses regarding PfGp96 regulation are therefore based primarily on sequence homology and comparative genomics rather than functional validation (Blatch, 2022; Muzenda et al., 2025).

### 4.3 ER-dependent and extracellular functions of Gp96 in immune modulation

Beyond its canonical ER-resident function, Gp96 can relocalize to the plasma membrane or be released extracellularly under conditions of cellular stress, inflammation, or tissue injury via non-canonical secretion pathways (Eletto et al., 2010; Samy et al., 2021). In this context, Gp96 no longer primarily acts as a folding chaperone but instead functions as a danger-associated molecular pattern (DAMP), engaging CD91 on antigen-presenting cells to promote antigen uptake, cross-presentation, and innate immune activation (Jockheck-Clark et al., 2010) (Figure 2).

Gp96 extracellular immunomodulatory activity reflects its intracellular role in immune receptor biogenesis (Pockley and Henderson, 2018). Plasma membrane profiling (PMP) combining SILAC and selective aminooxy-biotinylation identified 706 plasma membrane proteins with altered surface abundance in gp96-deficient versus reconstituted murine pre-B cells (Weekes et al., 2012). Beyond TLRs and integrins, several extended LDL receptor family members (LDLR, LRP6, Sorl1, LRP8) and CD180/Ly86 were shown to require Gp96 for proper surface expression (Staron et al., 2010). The enrichment of β-propeller and leucine-rich repeat motifs among these clients suggests shared structural determinants, supporting the concept of a Gp96-centered ER quality control module (Weekes et al., 2012).

Clinically, elevated circulating Gp96 levels have been reported in inflammatory conditions, including graft-versus-host disease and malaria, where concentrations correlate with disease severity (Seignez et al., 2017; Thiam et al., 2025) (Table 2). These observations reinforcing the association between extracellular Gp96 and inflammatory disease intensity, although a causal contribution to malaria pathogenesis remains unproven.

**TABLE 2:** Clinical and translational evidence linking extracellular or circulating Gp96 to disease severity.

Disease context	Study design	Cohort size	Biological matrix	Assay/Validation	Key findings	Level of evidence	Main limitations	Reference
Severe Plasmodium falciparum malaria	Hospital-based observational study	∼50–60 patients (severe vs. uncomplicated)	Plasma	ELISA (commercial kit; intra-assay CV reported)	Elevated circulating Gp96 in severe malaria; correlation with inflammatory cytokines	Direct human clinical evidence (moderate strength)	Moderate cohort size; cross-sectional; limited confounder adjustment	Thiam et al. (2025)
Graft-versus-host disease	Prospective clinical cohort	∼70 patients	Serum	ELISA + biochemical interaction assays	Gp96 correlates with disease severity and complement activation	Human clinical + mechanistic evidence	Non-malarial disease context	Seignez et al. (2017)
ER stress–driven inflammation	Experimental (human macrophages, murine models)	NA	Cell supernatant/tissue	Immunoblotting, ELISA, functional assays	Extracellular Gp96 regulates C3 cleavage and macrophage polarization	Strong mechanistic evidence	Preclinical; indirect relevance	Chaumonnot et al. (2021)
Autoimmune inflammation	Experimental + limited clinical samples	Limited	Serum	Immunodetection	Gp96 acts as a DAMP promoting innate immune activation	Conceptual evidence	No quantitative severity correlation	Weber et al. (2010)
Cancer	Multiple clinical cohorts	50–300+	Serum/tissue	ELISA, IHC	Circulating/surface Gp96 correlates with disease progression	Strong clinical evidence	Different pathology	Wu et al. (2016); Duan et al. (2021)
Viral infections	Review of experimental and clinical studies	NA	Serum/cell surface	Multiple	Gp96 contributes to inflammation and disease severity	Consistent indirect evidence	No malaria-specific cohorts	Xu and Blagg (2025)

Abbreviations: CV, coefficient of variation; DAMP, danger-associated molecular pattern; ELISA, enzyme-linkedimmunosorbent assay; IHC, immunohistochemistry; NA, not applicable.

Mechanistic studies outside malaria further identified complement component C3 as a key extracellular partner of Gp96. Under ER stress, co-secretion of Gp96 and C3 promotes cathepsin L-mediated C3 cleavage and macrophage polarization toward pro-inflammatory phenotypes (Chaumonnot et al., 2021). While this pathway links ER stress to inflammatory amplification, its specific relevance in malaria requires experimental validation.

### 4.4 Knowledge gaps and implications for host-parasite interactions

Although intracellular pathogens are known to exploit host chaperone systems, direct evidence that *P. falciparum* hijacks host Gp96 during infection is currently lacking (Blatch, 2022; Muzenda et al., 2025). Consequently, host-derived Gp96 functions in malaria should be interpreted cautiously and clearly distinguished from parasite-intrinsic processes.

Overall, existing data support a dual but mechanistically distinct role for Gp96 at the host–parasite interface: as an essential ER chaperone governing immune receptor biogenesis and stress responses in the host (Wu et al., 2012; Liu et al., 2020; Sharma et al., 2022), and as an extracellular stress-associated molecule functioning as a danger signal and correlating with inflammatory disease severity (Seignez et al., 2017; Chaumonnot et al., 2021; Thiam et al., 2025). Whether the parasite ER paralog PfGp96 fulfills analogous or divergent functions, and whether host and parasite Gp96-dependent pathways intersect functionally during infection, remain key open questions that warrant targeted experimental investigation.

## 5 Targeting Gp96 beyond malaria: mechanistic and translational lessons

### 5.1 Gp96 in viral and bacterial infections: defined host-pathogen interfaces

Work in infectious disease models supports context-dependent roles for Gp96 that are best understood through specific host pathways rather than a generic “stress chaperone” framing. In hepatitis B, Gp96 upregulation has been linked to host regulatory circuits that support viral replication programs, and Gp96 inhibition can reduce HBV outputs through defined host signaling dependencies (Fan et al., 2013; Qian et al., 2019; Xu and Blagg, 2025). In hepatitis C, Gp96-associated activation of inflammatory signaling, including NF-κB, has been reported to enhance profibrotic responses in infected hepatic systems, illustrating a role in host remodeling rather than direct viral protein folding (Jee et al., 2015). In herpesvirus infection, surface-localized Gp96 has been implicated in entry-related processes via interactions within receptor complexes (Prusty et al., 2014). For flaviviruses, Gp96-selective chemical probes can suppress replication *in vitro*, although the proximal molecular determinant (entry factor maturation vs. signaling dependencies) remains incompletely mapped (Rothan et al., 2019; Ma et al., 2020).

Bacterial studies provide a complementary angle by highlighting Gp96-dependent client specificity within host pathways relevant to invasion. For *Listeria monocytogenes*, Gp96 has been reported as a required host factor for productive infection in cellular models, consistent with selective co-option of host chaperone-dependent processes rather than universal pathogen reliance (Cabanes et al., 2005). While these mechanisms have not yet been demonstrated in *Plasmodium*, they provide a rationale for targeted malaria investigations focused on testing specific, mechanistically defined hypotheses, such as receptor biogenesis or endothelial and immune signaling pathways, rather than advancing broad claims that Gp96 functions as a universal infection factor.

### 5.2 Gp96 as a therapeutic target in cancer: selective biology and drug concepts

Gp96 is frequently dysregulated in cancer and can sustain tumor progression by supporting the maturation and surface expression of integrins, immune receptors, and growth factor signaling nodes, with associations to aggressive phenotypes and clinical outcomes (Yun et al., 2019; Duan et al., 2021). In breast cancer, Gp96 has been implicated in stabilizing HER2 signaling assemblies; pharmacological or antibody-based interference can promote receptor destabilization and tumor suppression *in vivo* (Li et al., 2015; Ansa-Addo et al., 2016; Alberti et al., 2022). In triple-negative breast cancer, membrane-associated Gp96 has been linked to macrophage polarization and invasive programs, and Gp96-selective inhibitors (e.g., PU-WS13) have been used to modulate these pathways in preclinical systems (Bouchard et al., 2021; Baverel et al., 2025). Beyond breast cancer, roles have been described in myeloma, and across solid tumors where Gp96 intersects AKT signaling, EMT, therapeutic resistance, and metastatic traits (Dejeans et al., 2012; Bedia et al., 2019; Duan et al., 2021; Pugh et al., 2022).

Collectively, findings from oncology provide a practical translational framework for exploiting Gp96-specific structural features to enhance selectivity, anticipating safety liabilities associated with critical client proteins, and designing robust target-engagement and pharmacodynamic strategies that may inform malaria-focused drug development.

### 5.3 Gp96 in autoimmunity and immune dysregulation

Beyond its role in cancer and infection, Gp96 has been implicated in autoimmune and immune-mediated inflammatory disorders, particularly when aberrantly expressed at the cell surface or released extracellularly (Tramentozzi et al., 2016; Guan et al., 2020). In systemic lupus erythematosus and rheumatoid arthritis, elevated extracellular Gp96 has been associated with activation of dendritic cells and macrophages, promoting sustained inflammatory cytokine production through CD91- and TLR-associated signaling pathways (Weber et al., 2010; Zhu and Lee, 2015). In murine arthritis models, blockade of Gp96-CD91 interactions attenuates joint inflammation without inducing broad immunosuppression, supporting the concept of pathway-selective immunomodulation (Zhu and Lee, 2015).

Gp96-dependent immune dysregulation also extends to graft-versus-host disease (GvHD), where ER stress-induced co-secretion of Gp96 and complement component C3 promotes macrophage polarization toward a pro-inflammatory phenotype via cathepsin L-mediated C3 cleavage and STAT1 activation (Seignez et al., 2017; Chaumonnot et al., 2021). These findings link ER proteostasis disturbances to systemic inflammatory amplification.

In addition, Gp96-peptide complexes have been explored as immunostimulatory platforms due to their capacity to enhance antigen cross-presentation and cytotoxic T cell priming (Strbo et al., 2013; Corigliano et al., 2020). While no Gp96-based formulations have yet received regulatory approval, this body of work underscores the dual nature of Gp96 in immune regulation: protective in controlled contexts, yet potentially pathogenic when dysregulated. These studies position Gp96 as a context-dependent regulator of immune homeostasis whose aberrant extracellular signaling may contribute to chronic inflammation and autoimmunity.

### 5.4 Implications for malaria research

Evidence from oncology, infectious disease, and immune-mediated disorders identifies Gp96 as a pharmacologically tractable regulator linking ER proteostasis with immune receptor biogenesis and inflammatory signaling (Hoter et al., 2018; Chaumonnot et al., 2021; Duan et al., 2021; Xu and Blagg, 2025). In the context of malaria, however, these findings should be interpreted as providing mechanistic hypotheses and translational frameworks, such as selective chemical scaffolds, target-engagement methodologies, and safety considerations, rather than direct evidence of host Gp96 exploitation by *Plasmodium* (Blatch, 2022; Muzenda et al., 2025). This cross-disciplinary foundation supports a structured research strategy in malaria that clearly distinguishes extracellular host Gp96 as a severity-associated biomarker (Thiam et al., 2025), intracellular host ER Gp96 functions essential for immune competence (Marzec et al., 2012; Seignez et al., 2017; Pugh et al., 2022), and parasite PfGp96 as a potential ER proteostasis target requiring rigorous functional validation (Posfai et al., 2018; Peng et al., 2021; Stofberg et al., 2025).

## 6 Gp96 as an antimalarial target: opportunities and challenges

Accumulating evidence supports PfHsp90 as an essential parasite chaperone and validates Hsp90 inhibition as an anti-plasmodial strategy in preclinical systems (Posfai et al., 2018; Adderley et al., 2020). However, the ER paralog PfGp96 remains comparatively under-characterized, and translation of Pf-directed Hsp90 targeting requires explicit attention to species selectivity, delivery to the parasite ER, and direct target engagement.

### 6.1 Chemical scaffolds targeting PfHsp90

PfHsp90 has emerged as a validated antiplasmodial target, and multiple chemical scaffolds have been developed to inhibit its ATP-dependent chaperone activity. The ansamycin geldanamycin (GA), the prototypical Hsp90 inhibitor, binds the N-terminal ATPase domain and disrupts client protein maturation (Mout et al., 2012). GA inhibits *P. falciparum* growth *in vitro*, including chloroquine-sensitive and -resistant strains, with reported effects across intraerythrocytic stages (Kumar et al., 2005; van Schalkwyk et al., 2010; Oluba, 2019). However, poor solubility and hepatotoxicity precluded clinical development. Semi-synthetic derivatives such as 17-AAG and 17-DMAG improved pharmacokinetics but retained limited parasite selectivity over host Hsp90 (Kitson and Moody, 2013; Murillo-Solano et al., 2017; Kitson et al., 2024; Xu and Blagg, 2025).

Structure-guided design and screening efforts subsequently generated second-generation inhibitors with improved potency. The β-carboline alkaloid harmine and optimized harmicine derivatives displayed nanomolar PfHsp90 inhibition and marked parasite selectivity, with compound 27a showing >1000-fold selectivity and activity against both hepatic and erythrocytic stages (Shahinas et al., 2013; Marinovic et al., 2023; Ahmad et al., 2024). Purine-based inhibitors such as PU-H71 also target the N-terminal ATP-binding pocket and exhibit antiplasmodial activity, although host cross-reactivity remains a challenge (Shahinas et al., 2013; Stofberg et al., 2021). Additional analogs identified through virtual screening (e.g., CP-7) demonstrate potent parasite inhibition with reduced cytotoxicity (Siddique et al., 2024).

Resorcyclic lactones such as radicicol and their stabilized derivatives further validate the N-terminal ATP pocket as a druggable site. Optimized radamides and isoxazole derivatives improve chemical stability and binding properties (Kitson and Moody, 2013; Stofberg et al., 2021). Luminespib (NVP-AUY922), a resorcinol-based inhibitor, exhibits differential affinity toward PfHsp90 isoforms, supporting scaffold refinement strategies (Magwenyane et al., 2022).

Beyond the N-terminal domain, the middle (MD) and C-terminal (CTD) domains represent alternative druggable regions. CTD-directed agents such as novobiocin and coumarin antibiotics, as well as MD-binding natural products, provide proof-of-concept for non-ATP-competitive inhibition (Gu (Goel et al., 2025; Gu et al., 2025). The recent structural resolution of full-length PfHsp90 now enables rational multi-domain inhibitor design to enhance parasite specificity (Gahlawat et al., 2025). These studies establish PfHsp90 as a chemically tractable target and demonstrate that isoform- and species-selective inhibition is achievable through structure-guided optimization, although host selectivity and *in vivo* validation remain critical translational challenges.

### 6.2 Insights from Gp96 inhibitor development

Gp96 has emerged as a promising therapeutic target in oncology and neurodegenerative disorders, in part because its pharmacological inhibition or genetic ablation in mammalian systems is compatible with limited cytotoxicity (Pugh et al., 2022; Xu and Blagg, 2025) (Table 3). However, the evaluation of Gp96-selective inhibitors in infectious diseases, and malaria in particular, remains limited (Duan et al., 2021; Dernovsek and Tomasic, 2023; Jia et al., 2025; Yan et al., 2025).

**TABLE 3:** Overview of selective Gp96 and PfGp96 inhibitors highlighting chemical scaffolds, mechanisms, and relevance for antimalarial drug development.

Chemical class/Inhibitor	Primary target	Mechanism of action	Selectivity/Activity	Strengths	Limitations	References
Radicicol	Gp96 (pan-Hsp90)	Binds N-terminal ATP pocket	Potent but non-selective	High affinity	Chemically unstable	Stofberg et al. (2024)
Radamides	Gp96	Stabilized radicicol analogs	Improved ER-targeting; enhanced stability	Better selectivity for Gp96	Requires malaria adaptation	Soga et al. (2003)
Resorcylic Isoxazoles	Gp96	Modified radicicol scaffold	Incorporates Gp96-selective pharmacophores	Improved drug-like properties	Limited biological data	Xu et al. (2021)
Resorcinol-based inhibitors (e.g., Luminespib—NVP-AUY922)	PfGp96 > PfHsp90	Binds ATP pocket with ER preference	∼10× higher affinity for PfGp96	Strong PfGp96 preference	Limited parasite-stage evaluation	Magwenyane et al. (2022)
Purine analogs (PU-WS13, PU-H36)	Gp96	Engineered to fit hydrophobic sub-pocket unique to Gp96	>200-fold isoform selectivity	High Gp96 selectivity	Not tested in malaria models	Bouchard et al. (2021); Khandelwal et al. (2018)
Aminoisoquinolines/Benzamides	Gp96	Exploit five–amino acid insertion in Gp96 ATP domain	Good solubility and PK	Tunable, selective scaffolds	No Plasmodium studies yet	Ardestani (2022)
NSC637153 (cp153)	Gp96 (client-binding)	Disrupts client folding/engagement; non-ATP competitive	Selective probe of Gp96-dependent functions	Unique mechanism; no Hsp70 induction	Not yet tested on PfGp96	Yan et al. (2025)
NECA	PfGp96	Interacts with PfGp96-specific residues (docking/MDS)	IC_50_ = 4.3–10 μM (*in vitro*)	First direct PfGp96-targeting hit	Moderate potency	Muzenda et al. (2025); Huck et al. (2019)

This table presents the principal inhibitors known to target Gp96 and its Plasmodium falciparum homolog PfGp96, including ATP-competitive and client-binding modulators. While many of these compounds were initially developed for cancer research, their selectivity profiles and mechanistic diversity offer valuable insights for antimalarial drug discovery. The table underscores the potential of ER-resident chaperones as underexplored therapeutic targets in malaria.

Structural studies identified a distinctive five-amino acid insertion within the N-terminal ATP-binding domain of Gp96 that generates a hydrophobic sub-pocket absent from cytosolic Hsp90 isoforms (Huck et al., 2019). Exploitation of this feature enabled the development of inhibitors with >200-fold selectivity for Gp96 (Alao et al., 2023). Subsequent optimization of resorcinol-, radamide-, and purine-based scaffolds, including PU-WS13 and PU-H36, improved specificity and pharmacokinetic properties while retaining potent antitumor activity (Khandelwal et al., 2018; Bouchard et al., 2021; Que et al., 2025). In parallel, NSC637153 (cp153), identified through a drGFP-based ERAD dislocation assay, functions as a selective probe of Gp96-dependent processes without inducing canonical Hsp90 stress responses, targeting client folding rather than ATP binding (Yan et al., 2025). Together, these advances provide a mechanistic framework that could be adapted to malaria drug discovery.

Comparative modeling further suggests that structural divergence between human Gp96 and its *P*. *falciparum* ortholog (PfGp96), particularly within the ATP-binding pocket, may allow species-selective inhibition (Yasir et al., 2025). Compounds such as luminespib and PU-WS13 have shown higher predicted affinity for PfGp96 than for the human ortholog (Monazzam et al., 2009; Bouchard et al., 2021), and computational strategies including active-learning docking and QSAR modeling may refine such scaffolds (Koirala et al., 2025; Valizadeh et al., 2025). NECA has also been reported to interact with PfGp96-specific residues *in silico* and to display moderate *in vitro* antiplasmodial activity (IC_50_ 4.3–10.0 μM), providing early proof-of-concept support for PfGp96 modulation (Huck et al., 2019; Muzenda et al., 2025). In addition, targeting extracellular Gp96 has been proposed as a complementary host-directed strategy to mitigate inflammatory pathology in severe malaria while preserving essential ER functions (Galluzzi et al., 2017; Pugh et al., 2022; Thiam et al., 2025).

Nevertheless, robust experimental validation of PfGp96 selectivity remains lacking. Current evidence largely derives from comparative sequence analysis, homology modeling, and docking studies rather than high-resolution structural characterization of PfGp96-ligand complexes (Stofberg et al., 2021). Although the canonical ATP-binding motifs are highly conserved across Hsp90 family members (Pearl and Prodromou, 2006; Hoter et al., 2018), comparative modeling studies of *P*. *falciparum* Hsp90 isoforms suggest potential differences in N-terminal domain topology and local pocket architecture (Monazzam et al., 2009; Shahinas et al., 2013; Blatch, 2022), which may influence ligand accommodation linker regions, and client-interaction surfaces suggest, but do not yet confirm, parasite-specific conformational states. Quantitative binding parameters and direct structural overlays comparing human Gp96 and PfGp96 are still unavailable, as high-resolution structures of PfGp96 in complex with small-molecule ligands have not yet been reported (Hoter et al., 2018). Accordingly, PfGp96 selectivity should be regarded as a promising but exploratory concept. Definitive validation will require integration of structural biology approaches, for example, X-ray crystallography or cryo-EM approaches, comparative ligand-binding analyses, and direct cellular target-engagement assays in infected erythrocytes before therapeutic claims can be substantiated (Shahinas et al., 2013; Challis et al., 2022; Molina-Franky et al., 2022).

## 7 Future perspectives and research directions

Evidence from oncology and immunology provides a mechanistic and pharmacological framework for PfGp96-directed antimalarial strategies (Ramdhave et al., 2013; Murillo-Solano et al., 2017; Rosenthal and Ng, 2020). Structure-guided design, molecular dynamics simulations, and scaffold optimization have enabled isoform-selective targeting of human Gp96 and should now be systematically adapted to the parasite ortholog (Khandelwal et al., 2018; Alao et al., 2023). However, translation to malaria requires explicit resolution of species selectivity, structural validation, and functional essentiality.

A primary priority is the rigorous discrimination between host Gp96 and parasite PfGp96. Although structural divergence has been predicted through comparative modeling (Monazzam et al., 2009), quantitative binding parameters and high-resolution structural overlays remain unavailable. Advances in cryo-electron microscopy, homology refinement, and biophysical profiling platforms now permit detailed mapping of isoform-specific features and should be leveraged to generate definitive selectivity data (Cebi et al., 2024; Rubach et al., 2025). Selective inhibition claims must therefore remain cautious pending comparative affinity measurements and direct cellular target-engagement validation (Challis et al., 2022; Jordan and Staack, 2023).

Functional validation of PfGp96 is equally critical. Conditional knockdown systems and CRISPR-Cas9-based approaches will be required to determine stage-specific essentiality and therapeutic vulnerability across liver and blood stages (Kudyba et al., 2018; Ishizaki et al., 2022). Parallel identification of PfGp96 client proteins will clarify its role in parasite proteostasis, stress adaptation, and protein export.

Immunological risk must also be explicitly addressed. In mammalian systems, Gp96 is indispensable for Toll-like receptor and integrin biogenesis (Marzec et al., 2012; Wu et al., 2012), raising safety concerns regarding systemic host inhibition. Selective PfGp96 targeting or modulation of extracellular Gp96-driven inflammatory signaling may represent safer alternatives (Wang et al., 2024).

Drug delivery represents an additional translational barrier. PfGp96-directed inhibitors must traverse the erythrocyte membrane, parasitophorous vacuole membrane, parasite plasma membrane, and ER lumen. Optimization of physicochemical properties and parasite-selective uptake mechanisms will therefore be essential (Varela et al., 2022).

Finally, therapeutic development requires direct demonstration of target engagement and *in vivo* validation. Cellular thermal shift assays (CETSA), chemoproteomics, and selective probe strategies should complement parasite growth assays to establish mechanistic causality (Jafari et al., 2014; Song, 2025). Humanized mouse models will be necessary to balance antiparasitic efficacy with host immune integrity (Angulo-Barturen and Ferrer, 2013; Tyagi et al., 2018). Concurrent biomarker development, including circulating Gp96 and ER stress signatures, may support pharmacodynamic monitoring and patient stratification (Ratna et al., 2021).

Collectively, while Gp96/PfGp96 represent compelling molecular nodes at the host-parasite interface, therapeutic translation will require structural confirmation of selectivity, genetic validation of essentiality, controlled immunological risk assessment, and robust pharmacological optimization.

## 8 Conclusion

Gp96 emerges from the current literature as a specialized ER-resident Hsp90 paralog that integrates proteostasis control, immune receptor biogenesis, and context-dependent inflammatory signaling. In mammalian systems, its non-redundant role in Toll-like receptor maturation, integrin folding, and ER quality control is firmly established. In parallel, extracellular Gp96 has been associated with immune activation and disease severity in inflammatory settings, including malaria, although a direct causal contribution to pathogenesis remains to be demonstrated.

In *P*. *falciparum*, the ER paralog PfGp96 represents a plausible but still insufficiently characterized component of parasite proteostasis. While structural divergence from the human ortholog suggests opportunities for species-selective targeting, robust quantitative binding data, high-resolution structural validation, and definitive functional genetic evidence are still lacking. Consequently, PfGp96 should currently be regarded as a promising exploratory target rather than a validated therapeutic entry point.

Cross-disciplinary insights from oncology, virology, and immune-mediated diseases provide valuable conceptual and methodological tools for malaria research, including selective scaffold design, target-engagement assays, and safety frameworks. However, successful translation to antimalarial therapy will require careful separation of host versus parasite mechanisms, rigorous demonstration of PfGp96 essentiality, and explicit management of immunological risk associated with host Gp96 modulation.

Taken together, Gp96 and PfGp96 occupy a strategic position at the intersection of ER stress, immune regulation, and host–parasite interaction. Advancing this field will depend on integrated structural biology, functional genomics, and pharmacological validation approaches capable of distinguishing mechanistic hypothesis from therapeutic feasibility.
